# Navigating an Automated Driving Vehicle via the Early Fusion of Multi-Modality

**DOI:** 10.3390/s22041425

**Published:** 2022-02-13

**Authors:** Malik Haris, Adam Glowacz

**Affiliations:** 1 School of Information Science and Technology, Xipu Campus, Southwest Jiaotong University, Chengdu 611756, China; 2 Department of Automatic Control and Robotics, Faculty of Electrical Engineering, Automatics, Computer Science and Biomedical Engineering, AGH University of Science and Technology, Al. A. Mickiewicza 30, 30-059 Krakow, Poland; adglow@agh.edu.pl

**Keywords:** artificial intelligent, end-to-end autonomous driving, safely navigation, conditional imitation learning (CIL), conditional early fusion (CEF), situation understanding, object detection, CARLA

## Abstract

The ability of artificial intelligence to drive toward an intended destination is a key component of an autonomous vehicle. Different paradigms are now being employed to address artificial intelligence advancement. On the one hand, modular pipelines break down the driving model into submodels, such as perception, maneuver planning and control. On the other hand, we used the end-to-end driving method to assign raw sensor data directly to vehicle control signals. The latter is less well-studied but is becoming more popular since it is easier to use. This article focuses on end-to-end autonomous driving, using RGB pictures as the primary sensor input data. The autonomous vehicle is equipped with a camera and active sensors, such as LiDAR and Radar, for safe navigation. Active sensors (e.g., LiDAR) provide more accurate depth information than passive sensors. As a result, this paper examines whether combining the RGB from the camera and active depth information from LiDAR has better results in end-to-end artificial driving than using only a single modality. This paper focuses on the early fusion of multi-modality and demonstrates how it outperforms a single modality using the CARLA simulator.

## 1. Introduction

Autonomous vehicles are essential to the future of the transportation industry. As a result, developing deep learning for autonomous vehicles is essential. No one should deny that recently, deep learning and computer vision have significantly impacted the automotive industry. Deep learning is used extensively in autonomous driving and augmented reality. Only a few of the complex and specialized functions needed for autonomous driving are automatic, for example, lane-keeping assistance, emergency braking (AEB) [1], active cruise control [2], forward collision warning (FCW) [3] and crash avoidance [4]. FCW and AEB are two of the first accident-avoidance features to be tested. The vehicle can only warn the driver of an impediment in front of them in FCW mode, and the driver must then determine whether to act. In AEB, however, as the vehicle approaches the front object, the vehicle begins to act by braking. As a result of integrating intelligent sensors such as Camera, LiDAR and Radar, ego-vehicles can make these decisions; however, low-quality sensors lead to many collisions and congestion. Modular pipelines (MPs) methods and end-to-end (E2E) learning methods are two common approaches for enabling self-driving to overcome these accident and alert the driver if some obstacle is found on the driving lane line.

MPs are used in the majority of autonomous vehicles [5,6]. Perception, route planning and control are three subproblems of the autonomous driving issue, that MPs divide into smaller, simpler subproblems. The method often depends on various sensors perfectly depicting the surrounding environment. Clear perception, tracking, mapping/localization, planning and control are the foundations of the MPs methods. This representation is then used to make a driving decision. While MPs are relatively easy to understand due to their modularity, they rely on complex intermediate representations that must be manually chosen (e.g., optical flow) and are often difficult to estimate with sufficient accuracy. Therefore, these methods may not be the best option for solving the sensorimotor control task. MPs also need significant quantities of labeled data, which may be difficult to come by, such as pixel-wise semantic segmentation [7,8] for neural network training, or high-definition maps for localization. Traffic sign recognition [9], obstacle detection [10,11,12,13], lane line recognition [14,15,16,17], monocular depth estimation [18,19,20], SLAM and positions recognition [21,22,23], and other sub-tasks, also become challenges for MPs methods.

In E2E approaches, deep neural networks are trained to directly produce the control outputs from raw sensor inputs. Imitation learning is the most popular approach [24]; it is a supervised learning approach based on human demonstrations. Using imitation learning to drive end-to-end has recently resurfaced as a topic of interest among academics. Imitation learning, like human learning, utilizes an image as an input and then predicts steering wheel angle, acceleration and deceleration values as outputs. End-to-end driving refers to the process of a model learning to drive from an expert demonstration, and it has been effectively used in lane-following [24,25] and obstacle avoidance off-road [26]. The article [27] argued that utilizing just an image as input is insufficient for determining whether the vehicle should turn left or right, or continue straight while approaching an intersection, and suggested a technique based on conditional imitation learning (CIL) and navigation commands to address this problem.

It is helpful to consider what factors an end-to-end driving system should include. We look at the issue from a variety of angles. The end-to-end driving system must start with a navigation command before following the selected route [27]. Besides this, we know it is helpful for human drivers to figure out what things are in front of the ego-vehicle. Xu et al. [28] have shown that combining a fully convolutional network (FCN) [29] with privilege learning may improve the driving model performance. Furthermore, human drivers pay close attention to the road. When a human driver sees that the traffic light has turned red, he will come to a stop, even if there are no other motorists or pedestrians in front of the ego-vehicle. Xu et al. [28] also show how segmentation information may help the model focus on the correct items. However, when the vehicle approaches an intersection, segmentation information may not be sufficient to allow the car to concentrate on valid objects, since the model cannot determine where to look and move at this moment without navigation commands. Intuitively, a branching design with a navigation command, in which various model portions handle distinct navigation circumstances, may be beneficial. In addition, human drivers can drive a vehicle safely since they can make out what class an object belongs to and its distance from the vehicle. We utilize an input RGB image and active depth information (D) from LiDAR to improve end-to-end artificial driving, rather than using only a single modality. Moreover, self-driving cars should be able to follow other vehicles. Using sequential images as input, instead of a single image, seems to have this impact. Long short-term memory (LSTM) [30] has been shown to improve driving performance in previous studies [28,31,32]. Finally, additional data, such as speed, are required in an end-to-end driving system, particularly when the speed restriction is considered.

Compared to the task in [27], our task parameters in this paper are slightly different. The traffic signal, other vehicles and pedestrians are all considered, but the speed limit is not. The model outputs include the vehicle steering angle and throttle, but the brake is not included. The brake is treated as a binary classification issue, with the vehicle either stopping or allowing driving at a steady speed. Our contributions to this study are to see whether utilizing multi-modality sensor data instead of depending on a single modality may improve the ability of an E2E driving model to assess and predict driving behavior. Color images (RGB) and depth (D) are considered single modalities; however, RGB-D is considered multi-modal. This work is built on the conditional imitation learning (CIL) [27] CNN architecture, which can take high-level commands. We explore RGB-D via early fusion of the single model, such as RGB image and depth (D). In addition, we use the CARLA simulator [33], as do many recent studies on E2E driving [34,35,36,37,38,39]. The remainder of the research paper is structured as follows:

- Section 2—Reviews the related work carried out and developed in the past few years.
- Section 3—Presents the CEF architecture for our proposed model using the CIL.
- Section 4 and Section 5—Summarize the experimental settings and the obtained results.
- Section 6—Discusses our conclusions and future work.

## 2. Related Work

Most research initiatives use MPs, which are the most common method of autonomous driving [40,41]. The perception stack must identify all elements of the traffic scene that are likely to be important for the driving choice, to construct the environmental model. Object detection [10,42], image segmentation [43,44] and motion estimation [45,46] are often trained and solved independently [47], with deep neural networks being used more recently for these tasks. This data may be compiled into an environment model [48,49], and a planning module creates an obstacle-free path for the control module to follow [50].

ALVINN [24] was the first imitation learning application of autonomous driving, predicting the steering angle from data from active and passive sensors. Deep learning advances have reignited interest in conditional imitation learning for autonomous driving [51]. ALVINN utilized a conditional order to display an E2E network for lanes following vacant highways, which monitored the steering angle from a single camera [52]. It learned longitudinal and transverse control through CIL, using a remote-control vehicle to execute route commands in a static environment [53]. Using numerous cameras and a 2D map for localization, it learned to navigate a road network. However, it relied on localization and route map generation and used drastically cropped images.

Pomerleau [20] and LeCun et al. [22] utilized ground vehicles and qualified deep networks to predict driver behavior using camera input. Bojarski et al. [25] show excellent results on real-world tasks, including highway following and flat-course driving. These studies focused on reactive tasks, like obstacle avoidance and lane following. However, the throttle and brake are not controlled, and lane and road changes are not considered, neither are go straight or slow down/stop maneuvers. In contrast, we propose a command-conditional formulation for more dynamic urban driving applications. Another difference is that the model is taught to control the steering angle as well as the throttle and brake, allowing it to drive itself. The decomposition of complex functions into simpler subtasks has been investigated from several perspectives.

Multiple layers of temporally extended subpolicies have been attempted using hierarchical methods to reinforce learning [54]. This kind of hierarchical breakdown is well exemplified by the choices framework [55]. This setting teaches fundamental motor abilities that may be used for various tasks [56]. For raw sensory input, hierarchical techniques were coupled with deep learning and utilized [57]. The main goal of these works was to simply profit from previous experience, and allow the hierarchical structure to uncover itself independently. This is a complicated and frequent issue, especially regarding sensorimotor skills. In addition, movement primitives have been used as building blocks in robotics to create complex motor skills [58,59]. A parameterized dynamical structure describes a simple motion, such as a strike or a throw, using movement primitives. On the contrary, the strategy we considered has more parameters and can solve more complex sensorimotor tasks, which combine perception and control. We focus on finding the next intersection or traffic light to reduce a vehicle’s speed and then making a left turn while avoiding dynamic obstacles, pedestrians, or other vehicles.

On the other hand, we use the CIL model to focus on early fusion and offer extra information about the expert objectives throughout the presentation. This article highlights the difficulty of learning and proposes a human policy that may be controlled. Hierarchical methods are like the concept of researching multi-functional and parameterized controllers. Parameterized targets are employed in the area of robotics [60,61,62]. A generalized reinforcement learning system with parameterized values transferred between states and goals, was proposed by Schaul et al. [63]. Koltun et al. [64] investigated families of parameterized objectives in the context of navigation in a 3D environment. Javdani et al. [65] looked at a scenario in which a robot assists a human and modifies his actions based on his evaluation. Although our study uses the same technique for training a conditional controller, the model architecture and application domain are different. Our method is on the E2E spectrum, but the controller comes with commands determining the driver intention and sensory input. That eliminates any uncertainty in mapping the perceptuomotor and provides a medium for communication that can guide the autonomous vehicle like a chauffeur.

Due to safety issues, driving simulators are primarily used in training and testing tasks. Open-source applications, such as TORCS [66,67,68] and Grand Theft Auto V (GTA V) [69,70], are popular driving simulators used for research. However, TORCS is not photorealistic or dynamic enough, since it lacks the essential elements of the scene, such as cross-roads, oncoming traffic, pedestrians, etc. GTA V is photorealistic, yet it is a closed source with limited customization and control over the environment. We utilize the recently released open-source simulator CARLA [33], which provides better customization of realism and flexibility, addressing some of the earlier simulators’ problems.

## 3. Methodology

We first outline the fundamental conditional imitation learning (CIL) network architecture, and then demonstrate how we integrate it with network information to exploit multi-modal perception data for early fusion.

### 3.1. Basic Conditional Imitation Learning Network Architecture

Consider each observation o=〈i;m〉 as containing an image ***i*** and a low-dimensional vector ***m*** which we refer to as measurements, following Dosovitskiy and Koltun [64]. A deep network represents controller ***F***. The network receives the image ***i***, the measurements ***m***, the command ***c***, and outputs the action ***a***. There are many ways to specify action spaces, such as discrete, continuous, or hybrid. Our driving experiments use 2D action spaces: steering angle and acceleration. In this case, the acceleration is negative, which represents braking or driving backward. The command c represents a category variable using a one-hot vector.

**Figure 1:**
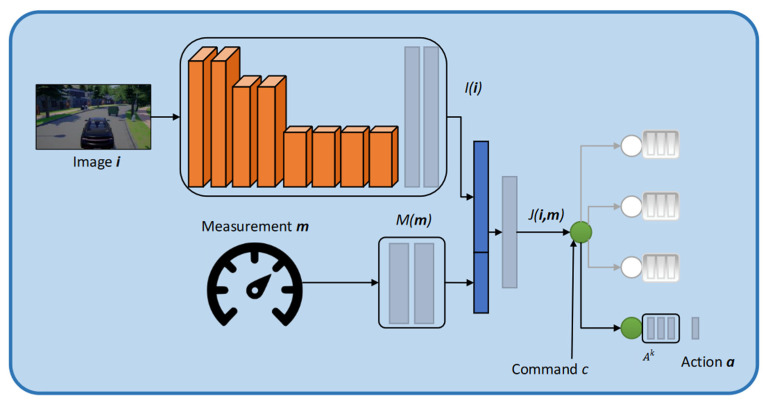
Fundamental conditional imitation learning (CIL) Architecture.

Figure 1 illustrates a basic conditional imitation learning architecture. The network takes the image, the measurements and the command as inputs. Each of these inputs is processed by its module: an image module ***I***(***i***), a measurement module ***M***(***m***) and a command module ***C***(***c***). Convolutional networks are used for the image module, and fully connected networks are used for the measurement module. The command module assumes a discrete set of $C=\left\{c^{0},\ldots ,c^{K}\right\}$ (including a default command $c^{0}$ corresponding to no specific command) and introduces a specialist branch $A^{i}$ for each of the commands $c^{i}$. The command ***c*** is a switch that selects which branch will be used at any given time. The network output is specified in Equation (1):

(1) $$F\left(i,\;m,\;c^{i}\right)=A^{i}\left(J\left(i,\;m\right)\right)$$

This type of architecture is known as branched. It is required that branches ***A^i^*** learn subpolicies that correspond to different commands. In a driving scenario, one module might focus on lane following, another on the right turns, and another on left turns. All modules are linked by their perception stream.

### 3.2. Early Fusion Multi-Model Network Architecture

**Figure 2:**
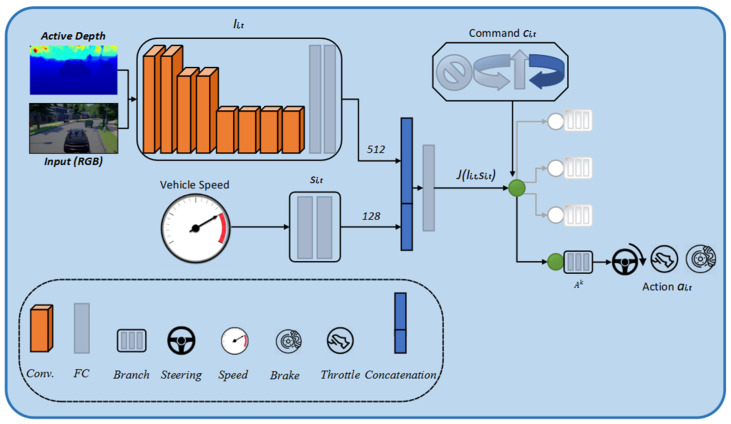
Early Fusion of Multi-modal Structure Diagram.

Our proposed architecture follows the conditional imitation learning model suggested in [27]. Figure 2 depicts how we fuse the input RGB image and active depth (***D***) information from the LiDAR in the early fusion method. $I_{i,t}$ consists of an RGB image of 200 × 88 pixels and 8 bits at each color channel, and the active depth (***D***) information from the LiDAR, $s_{i,t}$, signifies the current vehicle speed. The command $c_{i,t}$, and the output action $a_{i,t}$, defines three real-value signals that determine the next maneuver, such as steering angle, throttle and brake. The control command $c_{i,t}$ is introduced to handle complex scenarios, especially intersections, i.e., turn left, turn right, go straight, continue at the next intersection [27]. There are three continuous actions included in the action $a_{i,t}$, namely, the steering angle for the driving wheel $\left(a_{i,t}^{str}\right)$, the throttle setting $\left(a_{i,t}^{acc}\right)$, and the braking action $\left(a_{i,t}^{brk}\right)$. In order to clone human drivers’ driving behavior, we can learn a deterministic policy network ***F*** via conditional imitation learning. A set of four policy branches is specifically learned to encode the hidden knowledge for each case, which is then selected for use in action prediction. Controllable imitation learning aims to determine the parameters ***θ*** when the loss is optimal, and is defined as in Equation (2):

(2) $$\begin{array}{c} min\\ \theta \end{array}\overset{N}{\underset{i}{\sum }}\overset{Ti}{\underset{t}{\sum }}L\left(F\left(I_{i,t},c_{i,t},s_{i,t}\right),a_{i,t}\right)$$

The loss function L is defined as the absolute error between the three predicted actions ${\hat{a}}_{i,t}$ and the ground truth $a_{i,t}$ with the same command:

(3) $$L\left({\hat{a}}_{i,t},a_{i,t}\right)={\left|{\hat{a}}_{i,t}^{str}-a_{i,t}^{str}\right|}^{2}+{\left|{\hat{a}}_{i,t}^{acc}-a_{i,t}^{acc}\right|}^{2}+{\left|{\hat{a}}_{i,t}^{brk}-a_{i,t}^{brk}\right|}^{2}$$

Figure 2 depicts the network structure diagram, while Table 1 lists the specific parameters of the network structure. The network is composed of three parts. The first part consists of the eight convolutional layers, and two fully connected layers make up the feature extraction from the ***RGB-D***. In the convolutional layer, the kernel size is five in the first layer and three in the following layers. The first, third and fifth convolutional layers have a two-step stride. In the first layer, the number of channels is 32, increasing to 256 at the eighth layer in a convolutional neural network. The fully connected layers contain 512 units in each layer; this convolutional neural network layer is elaborated in Table 1. In the second part, the speed input is processed. It consists of two layers that are fully connected. The third part consists of four identical structures, each with three fully connected layers, in which the Conv2D layer contains a convolutional layer and a dropout layer, while the fully connected layer does not contain a dropout layer. The dropout layer randomly sets input units to zero with a frequency of rate at each step during training time, which helps prevent overfitting. A good value for dropout in a hidden layer is between 0.5 and 0.8. Here, the role of the hidden layers is to identify features from the input data and use these to correlate between a given input and the correct output. The neural networks have two main hyperparameters that control the architecture or topology of the network: the number of layers, and the number of nodes in each hidden layer. Thus, this proposed network will converge faster because it has fewer parameters to train.

### 3.3. A Pipeline of the Early Fusion of the Multi-Model Network

**Figure 3:**
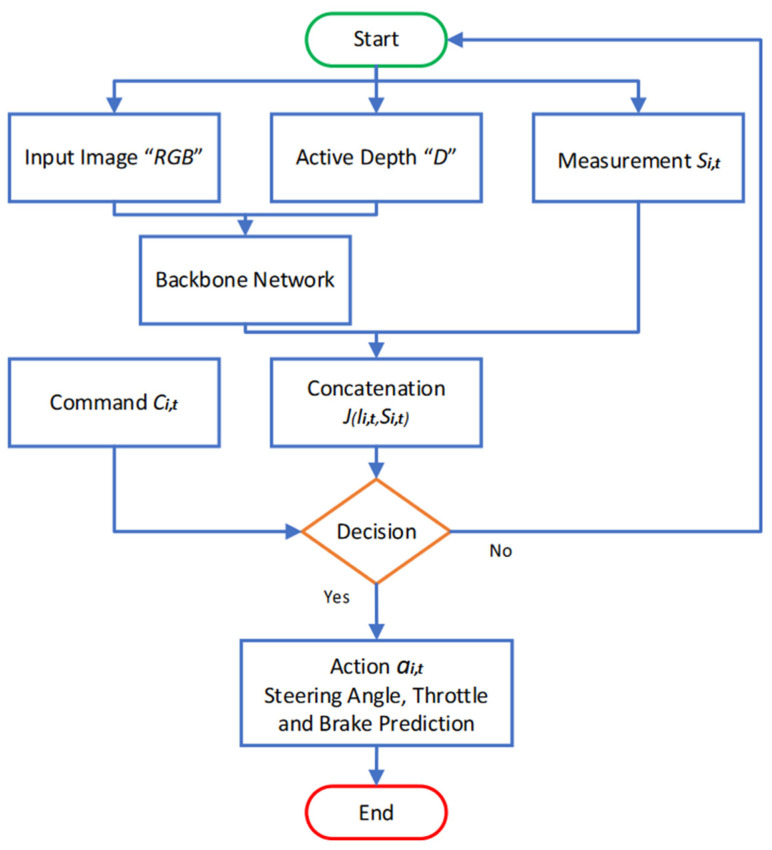
A Pipeline of the Early Fusion of the Multi-Model Network.

The whole pipeline is visually depicted in Figure 3. Our proposed algorithm follows the conditional imitation learning model suggested in [26]. Input $I_{i,t}$ consists of the fusion of the RGB image and the active depth (D) information from the LiDAR, $s_{i,t}$ signifies the current vehicle speed and the command $c_{i,t}$. In contrast to a single input mode, these multiple inputs are used for better end-to-end autonomous safety driving. In Figure 3, we can see that the convolution neural network block discussed in Section 3.2 is used to fuse RGB images with active depth (D) information from the LiDAR. Its help us to extract the most important information and associate that information with the measurement value, such as vehicle speed, $s_{i,t}$, by concatenating. This concatenating information is inserted into the decision part and the control command values. The control command $c_{i,t}$ helps in the complex scenarios, especially intersections, i.e., follow the lane, drive straight, turn left or turn right at the next intersection. The output action $a_{i,t}$ contains three continuous actions: the steering angle for the driving wheel $\left(a_{i,t}^{str}\right)$, throttle setting $\left(a_{i,t}^{acc}\right)$ and braking action $\left(a_{i,t}^{brk}\right)$.

## 4. Experiments

The training, validation and testing of the model proposed in this paper were performed using the TensorFlow [71] framework and cuDNN [72] kernel. The hardware equipment included a workstation with Intel@CoreTM i7-6800K CPU@3.40GHz and a GTX 1080Ti graphic card.

### 4.1. CARLA Simulator

Our end-to-end model was trained and tested in an open-source simulation environment for ease of use and safety. The simulation environment was chosen mainly to save data collection and labeling time. CARLA [33] is a state-of-the-art vehicle controller simulator that lets users develop their own vehicle controllers with RGB and depth cameras, and LiDAR sensors. The data about the car, such as speed, steering angle, throttle positions and brake positions, are available in simulations and the data about the environment include lane lines and traffic signs. CARLA offers more information about urban towns with different layouts. Other simulators, such as Udacity and TORCS [73], are not designed for urban area driving. Intersections, lane rules and other complexities are lacking, such as differentiating between urban driving and highway driving.

**Figure 4:**
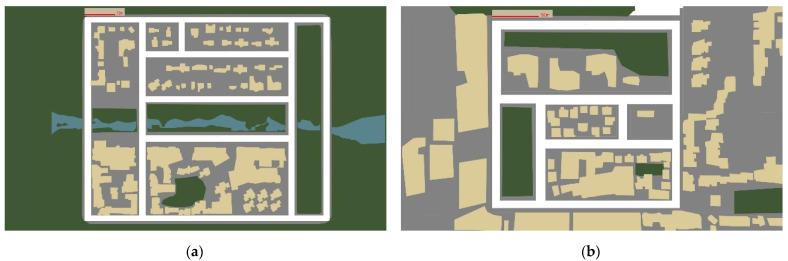
Aerial viewpoints of (**a**) Town 1 and (**b**) Town 2.

It is important to collect appropriate data to achieve imitation learning. The simulation environment is based on CARLA, with Logitech G29 Driving Force Racing Wheel for driver input, and FFB Checker for force feedback. As shown in Figure 4, CARLA provides maps and sample views of Town 1, used for training and Town 2, used exclusively for testing. Town 1 has 2.9 km of road and 11 intersections, while Town 2 has 1.4 km of road and eight intersections. It provides a professionally designed environment with buildings, vegetation and traffic signs, as well as vehicular and pedestrian traffic. Before data collection, we redefined the scope of the steering angle. The Logitech G29 Driving Force Racing Wheel supports a rotation angle of [−900,900], the same as a real car, but in the CARLA simulator, the steering wheel angle is set to −1 or 1.

We gathered the driving behavior data of a human driver under Left, Right, Follow, and Straight instructions (in the acquisition, the front car was a Lincoln MKZ 2017, made available by CARLA). During this time, the vehicle’s speed at the time, the RGB camera frame data from the front-view camera, and the active depth information from the LiDAR, were also collected. We set the camera at the same position and parameters (such as FoV, resolution and type) as [27]. In all commands except the Follow command, autonomous vehicles had to avoid obstacles. Therefore, we collected data to reduce the risk of collision with vehicles and pedestrians in front of Right, Left and Straight.

### 4.2. Data Collection

**Figure 5:**
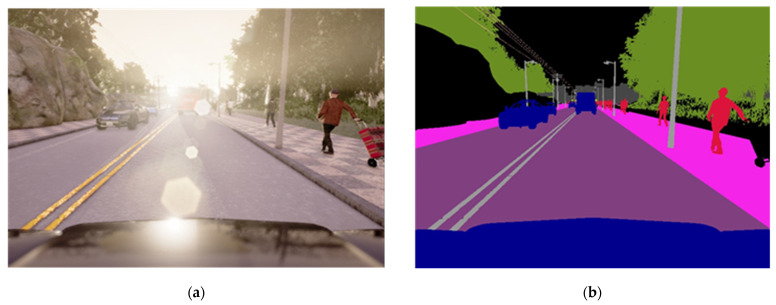

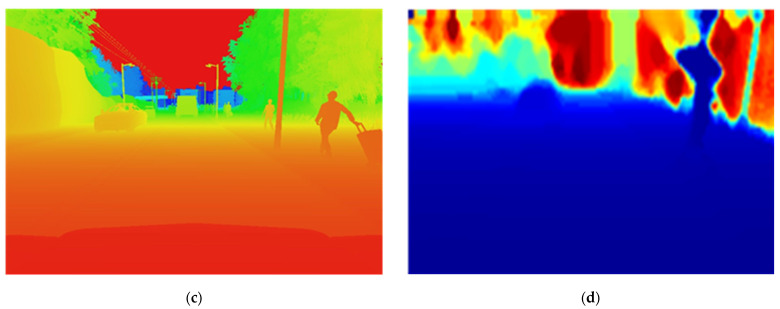
Images utilized in our proposed model. (**a**) Input RGB image, (**b**) Semantic Segmentation Ground Truth, (**c**) CARLA depth ground truth, (**d**) Post-processed image of an active depth.

The collected data set is the same as [74], since the driving time was 25 h in Town 1, by balancing different weather environments. In short, this dataset was generated by a hard-coded self-pilot with full access to all CARLA-sensitive driving data. When traveling on a straight road, the autopilot maintained a steady speed of 31.8 km per hour, slowing down or stopping at the next intersection before turning. The raw data that we processed and synchronized at 10 fps includes the following input details:

1. The center front camera and the two side cameras at 30° left and right. The only camera utilized for autonomous driving is the one in the center. In a self-driving vehicle, the images from the side cameras are solely used during training to mimic the driving error recovery graph [25]. The collection includes 2.5 million RGB pictures with a resolution of 800 × 600 pixels and relevant ground truth. The RGB input image is cropped to eliminate the sky and extremely near region, then scaled to provide a channel resolution of 200 × 88 pixels in our early fusion model. Data augmentation is essential for good generalization in our initial experiments. During network training, we conduct augmentation online. We add a random subset of transformations of random sampling magnitudes to each image to be presented to the network. Changes in brightness, contrast, lighting and Gaussian noise are all part of the transformation. Gaussian blur, salt and pepper noise, and area dropout are some of the effects available. Geometric improvements, such as translation and rotation, are not performed, since the control command is not invariant to these transformations.
2. We develop an upper bound driver using perfect semantic segmentation in this work. In order to develop this upper bound, the twelve semantic classes of CARLA are mapped to five, which we consider sufficient. We keep the original road surfaces, vehicles and pedestrians, while lane markings and sidewalks are given the status of ‘lane limits’ (Towns 1 and 2 only have roads with one go and one return lane, separated by continuous double lines), and the remaining seven classes are given the status of ‘other’.
3. Premebida et al. [75] obtained depth data from the LiDAR point cloud, and we think RGB images include dense depth data. The CARLA depth of ground truth comes straight from the Z-buffer used for displaying the simulation, and it is extremely accurate. The depth value is 24-bit encoded and spans from 0 to 1000 m, implying a depth precision of around 1/20 mm. An active sensor range coverage and depth accuracy significantly exceed a passive sensor. The use of depth data has been post-processed to make it more realistic. The Velodyne information from the KITTI dataset [76] provides a realistic sensor reference. First, we reduce the depth value only to examine pixels inside the 1100-m range, i.e., pixels with outside values in the depth image. On the other hand, this range does not contain any depth information. Second, we re-quantify the depth value within 4cm of the original. Third, we fix the pixels that have no data. Finally, we apply a median filter to prevent establishing precise depth boundaries between objects. New depth images are utilized during training and testing. Figure 5 shows an example of a CARLA depth image and the equivalent post-processed version.

### 4.3. Training

Given the autopilot ground truth values, the networks are trained to minimize the mean-squared error of the steering angle, throttle and brake values. We follow the multi-tasking training approach where we have multi-tasks for four corresponding network heads for left-branch, right-branch, straight-branch, and slow down for the speed/stop-branch. According to the selected command, the loss is minimized for the current active head when one head is activated. The network is trained for 200 epochs with a batch size of 16. For optimization, the ADAM optimizer [77] is used where β1 = 0.9 and β2 = 0.99, and there is an initial learning rate of α = 0.0002.

## 5. Experiment Results

### 5.1. Success Rate Comparison with Previous Methods

We compared our proposed model to previous single-modality methods discussed in the related work section, to assess its superiority further. This comparison is entirely based on the CARLA benchmark results because not all relevant papers offer comprehensive training techniques or training datasets. The benchmark test included six different driving activities with increasing complexity, including driving in a straight line, driving through a single turn, navigating through the town, taking several turns, and navigating through the town with random obstacles, such as vehicles, obstacles and pedestrians, among others. The agent starts at a predetermined place in town and completes a task within a certain amount of time. The time restriction is equivalent to the time it takes to travel 31.8 km per hour on the best route to the destination. We compared our conditional early fusion (CEF) method to the listed models in Table 2. These results are reproduced from the following manuscript listed in Table 2.

**Figure 6:**
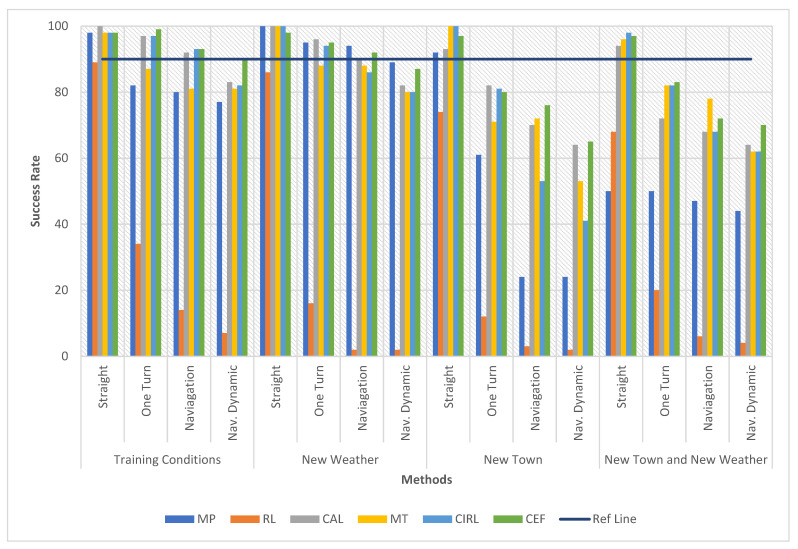
Success Rate Comparison with Previous Methods.

The comparison results are shown in Figure 6, with a reference line for a clear values analysis. In the face of a complicated traffic situation, the early fusion of the RGB image and the active depth method had a better success rate on the CARLA benchmark. On the other hand, this early-fusion technique may be used in combination with the CAL and CIRL procedures, which are highly compatible. This comparison to prior studies backs up our assertion that multi-modality may aid E2E driving. As shown by Figure 6, our proposed model performed better than nearly all the tasks, except for the training town task under changing weather conditions, where modular perception performed slightly better. In contrast, we can also see that the results of reinforcement learning can vary greatly. Reinforcement learning was trained using more data; 12 days of driving, compared with 14 h for imitation learning. A second explanation is that urban driving is more difficult than most tasks, especially asynchronous reinforcement learning. Moreover, a driving scenario is more complex than reinforcement learning maze navigation, for instance, because the agent needs to deal with vehicle dynamics and visual perception in a cluttered dynamic environment.

According to the benchmark, towns and weather conditions are divided into four models, under each of which the four driving tasks have to be tested in 25 episodes. These are the four models relating to towns and weather in the benchmark:

➢ Training conditions: driving (i.e., running the episodes) in the same conditions as the training set (Town 1, four weather conditions).
➢ New Town: driving under the four weather conditions of the training set but in Town 2.
➢ New Weather: driving in Town 1 but not seen at training time under the two weather conditions.
➢ New Town and Weather: driving in conditions not included in the training set (Town 2, two weather conditions).

### 5.2. Performance of Single & Multi-Models

The suggested model performance against the original CARLA benchmark is shown in Table 3. We incorporated a model trained in perfect semantic segmentation (SS) based on the five classes examined here for autonomous driving, as illustrated in Figure 5. Therefore, we think of this model as an upper limit. Its average production is ≥96%, and it has reached 100% on numerous occasions. Table 2 shows that our suggested multi-model may drive correctly in CARLA if we give appropriate input. Indeed, we can observe that active depth provided complete information for E2E driving, and that its performance in an untrained context is considerably superior to RGB. In most instances, however, multi-modality (RGB-D) performs better than utilizing just one model, such as RGB or simply D. The most apparent scenario is a new town and weather conditions with dynamic objects, i.e., under challenging circumstances. Single modality of RGB can obtain a success rate of 53.5%, D alone 71.62%. At the same time, the early fusion of multi-modality achieved a success rate of 94.3%. The primary issue we aimed to address in this article, is whether the early fusion of multi-modality may enhance the performance of conditional imitation learning over a single model.

### 5.3. Grad-CAM Visualizations

**Figure 7:**
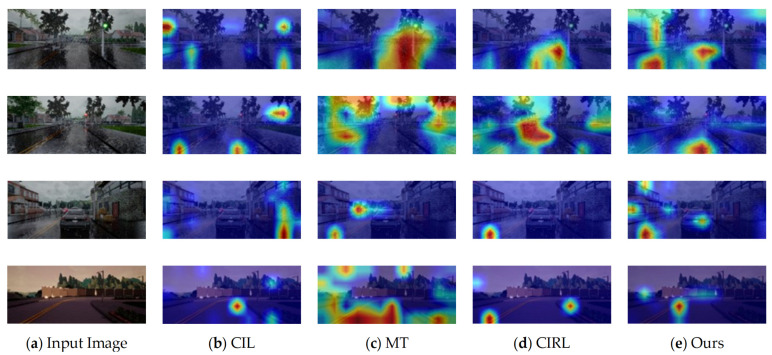
Qualitative Results Analysis by Grad-CAM Visualization.

Figure 7 presents a Grad-CAM visualization of different methods using random samples from training conditions for qualitative analysis [78]. Using Grad-CAM, we can investigate what aspects of the input significantly affect the output. Our first step was to visualize the salient parts of the structure that contributed to control output. Figure 7 shows how Grad-CAM attention maps cover useful regions, such as the center line or the border between the road and sidewalk. Our proposed method looks at narrow, yet important, points, such as the center of the road and the leading vehicle, while CIL might highlight irrelevant information and MT appears to be distracted by large regions. We visualized attention maps based on traffic light predictions as a second step. MT and CIRL widely capture the traffic light, including the pole, when it is red, whereas our proposed approach attentively looks at it, but the red color point is not included.

### 5.4. Prediction of Steering Wheel Angle

**Figure 8:**
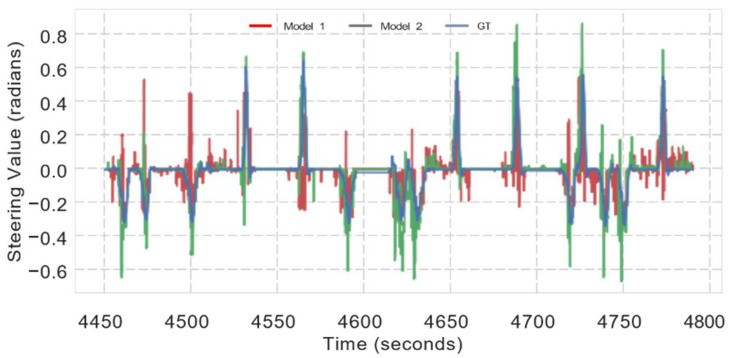
Detailed evaluation of two driving models with the prediction of steering wheel angle.

To further analyze our proposed model goodness, we compared it to predicting the steering wheel angle using single and multi-modality methods. The mean square error (MSE) loss and forward-facing camera were used to train the first model. The MSE loss and RGB and depth fusion were utilized in the second model (D). The steering wheel angle was standardized between −1.0 and +1.0, as illustrated in Figure 8, with positive values indicating right-side rotation, and negative values indicating left-side rotation. We used the linear transformation Equation (4) to normalize the value:

(4) $$\theta_{normalized}=-0.5+max\left(0,\;min\left(1.0,\frac{\theta_{raw}-\theta_{min}}{\theta_{max}-\theta_{min}}\right)\right)$$

where, $\theta_{normalized}$ is the normalized steering angle between −1 and +1; $\theta_{raw}$ is the actual steering wheel angle (in radians) measured from the vehicle; $\theta_{max}$ and $\theta_{min}$ represent the maximum and minimum steering wheel angle, respectively. Similarly, we normalized throttles values between −1 and 1.

The ground truth steering signal over time is presented in the blue line. Figure 8 shows the predictions of the basic CIL model (Model 1) and the early fusion of the multi-model (Model 2) in red and green, respectively. The model predictions have a significant qualitative difference: both often depart from the ground reality.

### 5.5. Path Planning

**Figure 9:**
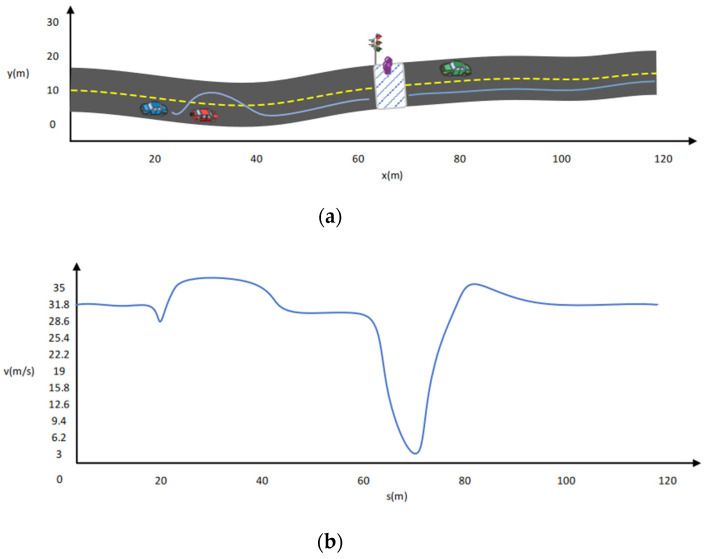
Dynamic path planning results in a straight road with multiple challenges. (**a**) Vehicle overtakes the vehicle in front and waits for the green signal, (**b**) Speed Curve.

**Figure 10:**
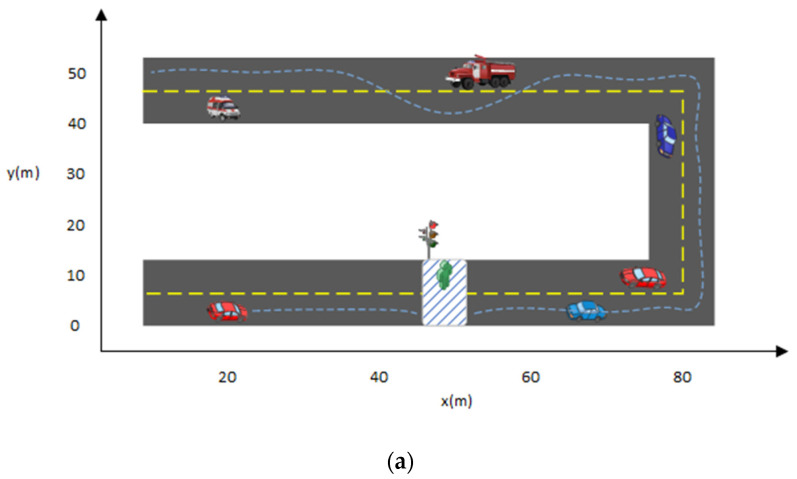

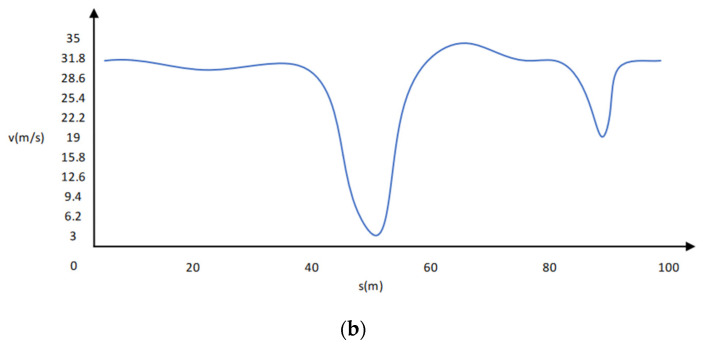
Dynamic path planning results for the U shape road with multiple challenges. (**a**) Vehicle overtakes the vehicles in front, takes two left turns, and waits for the green signal, (**b**) Speed Curve.

Specifically, here are the results include waiting for pedestrian crossings and overtaking a vehicle in front. There is a predefined map with vehicles and pedestrians on it. Cubic splines describe the road edges. White dashed lines indicate the center lines of these roads. A zebra crossing is represented by the rectangle box with white and blue lines on the road. The yellow dash lines show the vehicle path. Figure 9 and Figure 10a,b show path-planning moments, overall trajectories, and the speed graph of the vehicle, respectively. Here ***s*** and ***v*** are the shift and speed. The speed graph in this paper represents total values, which account for many factors, including decreasing speed, throttling, and air and ground resistance. Figure 10b shows the first left turn effect of the road on the second curve. Meanwhile, the blue car curve effect does not appear on the x-axis because it is also moving, and its curve does not exit under 120 m once the agent crosses it.

## 6. Conclusions

This paper presents a comparison of single- and multi-modal perception data for end-to-end driving. We focused our analysis on the RGB and depth data, since they are usually available in autonomous vehicles through cameras and active sensors, such as LiDAR. In this study, we examined the driving performance of single-modal CIL, MP, RL, CAL, MT and CIRL models, as well as multi-modal CIL (RGB, depth) models according to early fusion paradigms using the CARLA simulation environment. The depth information available in CARLA is post-processed in order to obtain a more realistic range of distances and depth accuracy. This depth is also used to train a depth estimation model so that the experiments cover multi-modality, not only from a multisensory perspective (RGB and active depth), but also from a single-sensory perspective (RGB and estimated depth). Our results show that the multi-modal CIL models significantly improve performances in all scenarios, especially in “New Town” and “New Town and New Weather” compared to the single models.

There are a number of limitations to the method presented here that we believe are essential to resolve, on order to achieve (and exceed) human-level driving. This means that the user cannot access long-term dependencies and cannot make sense of the road scene. In addition, this method lacks a long-term planning model that is important for safely interacting with occluded dynamic agents. There are many promising directions for future research. The time and safety measures needed for a closed-loop policy are major limitations; thus, robustly evaluating a policy offline and quantifying its performance is a critical research area.

In the future, a video might be used to provide temporal information as well. In this way, the end-to-end driving model is able to distinguish between dynamic and static objects more precisely. Furthermore, it would be interesting to see if the proposed method could be applied to real-world data, such as the BDD100K dataset [79], then to demonstrate how a real car would behave under our control. We could also try to improve the model’s ability to avoid collisions by including other useful information. In urban areas, it is possible to achieve autonomous driving at the L4 level or even at the L5 level with simple navigation instructions if the generalization abilities of this model are strong enough.

## Figures and Tables

**Table 1:** Network Details.

Layer	Output Shape	Layer	Output Shape
Input Image	88×200×3	Input Speed	1
Conv2d_1	42×98×32	FC	128
Conv2d_2	40×96×32		
Conv2d_3	19×47×64		
Conv2d_4	17×45×64		
Conv2d_5	8×22×128		
Conv2d_6	6×20×128		
Conv2d_7	4×18×256	FC	128
Conv2d_8	2×16×256		
Flatten	8192		
FC	512		
FC	256		
FC	256		
FC	256	Total 4 branches for all 4 commands	
FC	256		
Output	1		

**Table 2:** Result Reproduction Details.

Model	Abbreviation	Manuscript
Modular Perception	MP	[33]
Reinforcement Learning	RL	[33]
Controllable Imitative Reinforcement Learning	CIRL	[35]
Multi-Task Learning	MT	[36]
Conditional Affordance Learning	CAL	[37]

**Table 3:** Mean and Standard Deviation of Success Rate on the Original CARLA Benchmark.

			Active		Estimated				Active		Estimated	
Task	SS	RGB	D	EF	D	EF	SS	RGB	D	EF	D	EF
	Training Conditions						New Town					
Straight	98.0	96.3	98.7	98.3	92.3	97.3	100	84.0	94.3	96.3	78.3	71.6
One Turn	100.0	95.0	92.0	99.0	84.6	96.3	96.6	68.0	74.3	79.0	46.3	47.0
Navigation	96.0	89.0	89.3	92.6	75.3	94.3	96.0	59.6	85.3	90.0	45.6	46.6
Nav. Dynamic	92.0	84.0	82.6	89.3	71.0	90.6	99.3	54.3	70.3	84.3	44.3	46.6
	New Weather						New Town and Weather					
Straight	100.0	84.0	99.3	96.0	92.0	84.6	100	84.6	97.3	97.3	78.0	89.3
One Turn	100.0	76.6	94.6	94.6	93.3	80.6	96.0	66.6	72.7	82.7	62.6	64.0
Navigation	95.3	72.6	89.3	91.3	73.3	80.6	96.0	57.3	84.0	92.7	55.3	60.7
Nav. Dynamic	92.6	68.6	90.0	86.0	76.6	77.3	98.0	46.7	68.3	94.0	54.0	49.3
